# Automatic analysis algorithm for acquiring standard dental and mandibular shape data using cone-beam computed tomography

**DOI:** 10.1038/s41598-018-31869-6

**Published:** 2018-09-10

**Authors:** Jae Joon Hwang, Sang-Sun Han, Chena Lee, Yun-Hoa Jung

**Affiliations:** 1 DDS., PhD., Department of Oral and Maxillofacial Radiology, School of Dentistry, Pusan National University, Yangsan, Korea; 2 DDS., PhD., Department of Oral and Maxillofacial Radiology, Yonsei University College of Dentistry, Seoul, Republic of Korea

## Abstract

This study aims to introduce a new algorithm developed using retrospective cone-beam computed tomography (CBCT) data to obtain a standard dental and mandibular arch shape automatically for an optimal panoramic focal trough. A custom-made program was developed to analyze each arch shape of randomly collected 30 CBCT images. First, volumetric data of the mandible were binarized and projected in the axial direction to obtain 2-dimensional arch images. Second, 30 patients’ mandibular arches were superimposed on the center of the bilateral distal contact points of the mandibular canines to generate an average arch shape. Third, the center and boundary of a panoramic focal trough were obtained using smoothing splines. As a result, the minimum thickness and transition of the focal trough could be obtained. If this new algorithm is applied to big data of retrospective CBCT images, standard focal troughs could be established by race, sex, and age group, which would improve the image quality of dental panoramic radiography.

## Introduction

Panoramic radiography is a widely used imaging modality in the routine dental examination. Since the head and neck area contains complex and important structures and panoramic radiography is used as the first diagnostic tool to screen diseases, it is important to obtain adequate image quality^1,2^. This technique produces a tomographic image using a specific 3-dimensional (3D) curved zone or focal trough (image layer), in which the structures are reasonably well defined^3^.

Although diverse arch shapes and dimensions have been reported according to race, sex, and age groups^4–7^, little has been reported about average arch shapes. Therefore, the focal trough, which is constructed according to the average arch shape, varies across brands of equipment^8–10^. Furthermore, neither the data collection criteria of each manufacturer nor the method of creating the average arch shape and focal trough is publicly available. Lack of agreement in the focal trough can be an obstacle to the standardization of panoramic radiography, and the consequent variation in images may affect the diagnostic accuracy in multiple ways^11,12^. First, different magnification across brands results in different measurements of the same anatomical structures. As many dentists still rely on panoramic radiography for implant planning, the possibility of misjudging the horizontal and vertical length of the bone or nerve structures might increase the risks associated with implant surgery^13^. Second, differences in image distortion and ghost image formation can mask an existing pathology^14^. This can also increase the possibility of missing an existing lesion or the postoperative recurrence of a lesion. Furthermore, the non-unified panoramic focal trough has become a stumbling block in the development of standard phantoms for regular image quality assessment of panoramic radiography. Therefore, it is necessary to obtain the average arch shapes of each race, sex, and age group and to integrate that information with the standard focal trough acquired using commercial panoramic radiography equipment in order to obtain reliable image quality.

A previous study compared the average arch shapes of 3 ethnic groups using 2-dimenstional (2D) submentovertex radiography^15^. Their method took time and effort because it required manual drawing of the center of the mandible and determination of about 50 points. They also used a small amount of data (35 patients) to represent each sex and ethnic group. Other studies used only 12 to 18 points to obtain the central curve of the dental arch^16,17^. For processing big data to acquire a standard arch shape from retrospective 3D cone-beam computed tomography (CBCT) images, which have great dimensional accuracy and reliability^18^, the development of an automatic algorithm is necessary.

The purpose of this study is to introduce a new and convenient algorithm for obtaining an average dental and mandibular arch shape using retrospective CBCT data, which can be used in a big data study to establish standard focal troughs for panoramic radiography.

## Materials and Methods

### Ethics Statement

This study was conducted with the approval of our Institutional Review Board (IRB) (2-2015-0044) of our university dental hospital. This study had a retrospective design and all data were analyzed anonymously. The IRB of our dental hospital waived the need for individual informed consent. This study is HIPAA compliant and all methods were performed in accordance with the relevant guidelines and regulations in our dental hospital.

### CBCT scans

CBCT data were randomly collected retrospectively from 30 patients (Table 1) who underwent CBCT due to clinical problems such as impacted third molar contacting the mandibular canal and temporomandibular disease between January 2015 and December 2016 in our university dental hospital, but were shown to have no pathologic bone changes in either condylar head. Patients with surgical defects, missing teeth or implants, or dental or skeletal malocclusion were excluded. The CBCT images were obtained by trained technicians, with the occlusal plane parallel to the floor and the mid-sagittal plane perpendicular to the floor. An Alphard 3030 (Alphard Roentgen Ind., Kyoto, Japan) apparatus was used to obtain CBCT images with exposure conditions of 80 kV and 5 mA with a 154 mm × 154 mm field of view.

**Table 1:** Sex, number, and age of patients.

	Number of patients	Age (mean ± SD)
Male	11	20.91 ± 6.39
Female	19	29.26 ± 11.97

### Image processing

**Figure 1:**
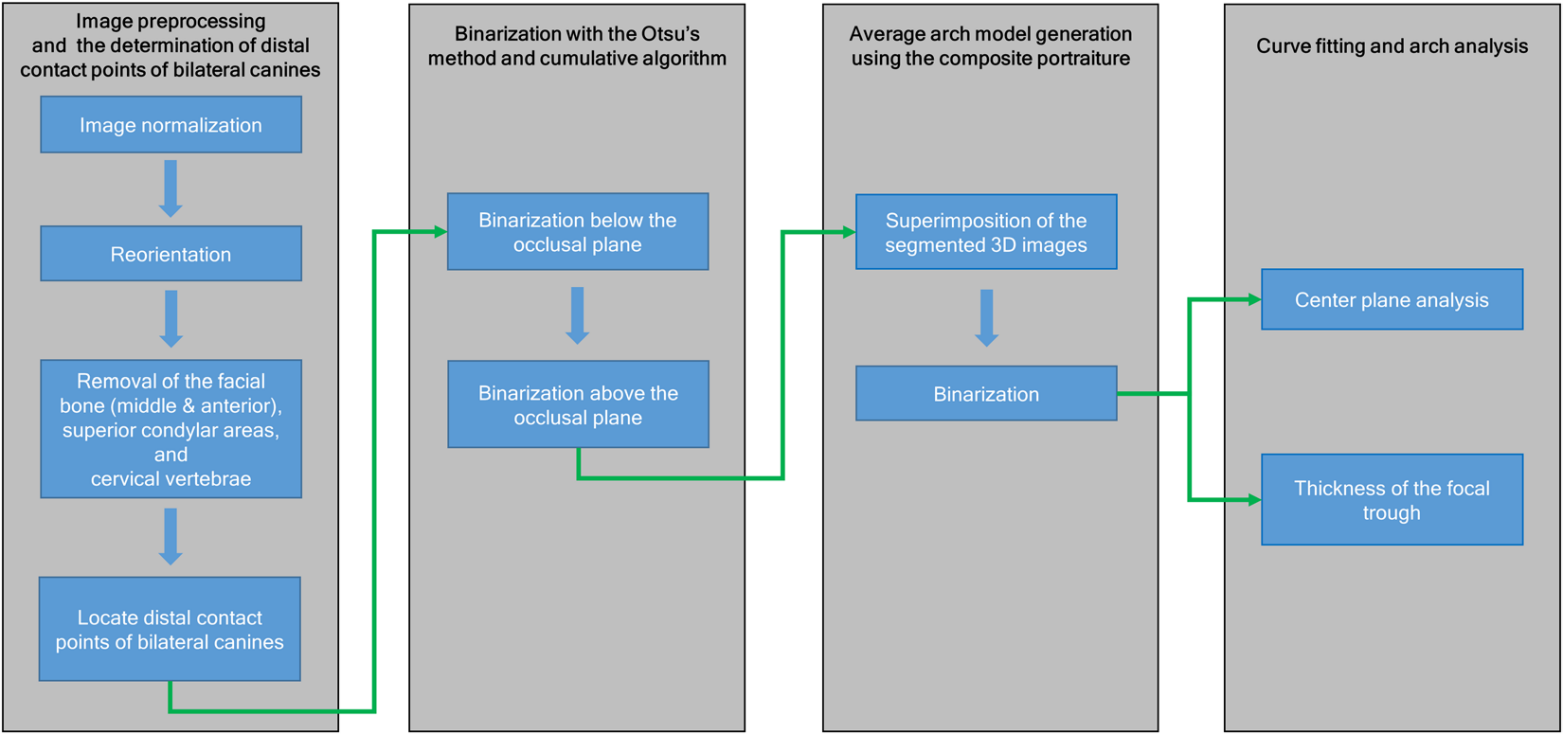
Flowchart of image processing and analysis. After image preprocessing and determination of the distal contact points of the bilateral canines, binarization and average arch shape generation were performed. The center, boundaries, and minimum thickness of the focal trough were then analyzed.

A customized computer program was made using MATLAB 2016a (MathWorks, Natick, MA, USA) and used for the generation and analysis of the average arch shape. Two dentists processed images twice at 2-week intervals. Figure 1 shows the flowchart of the image processing and analysis.

#### Image preprocessing and determination of the distal contact points of the bilateral canines

**Figure 2:**
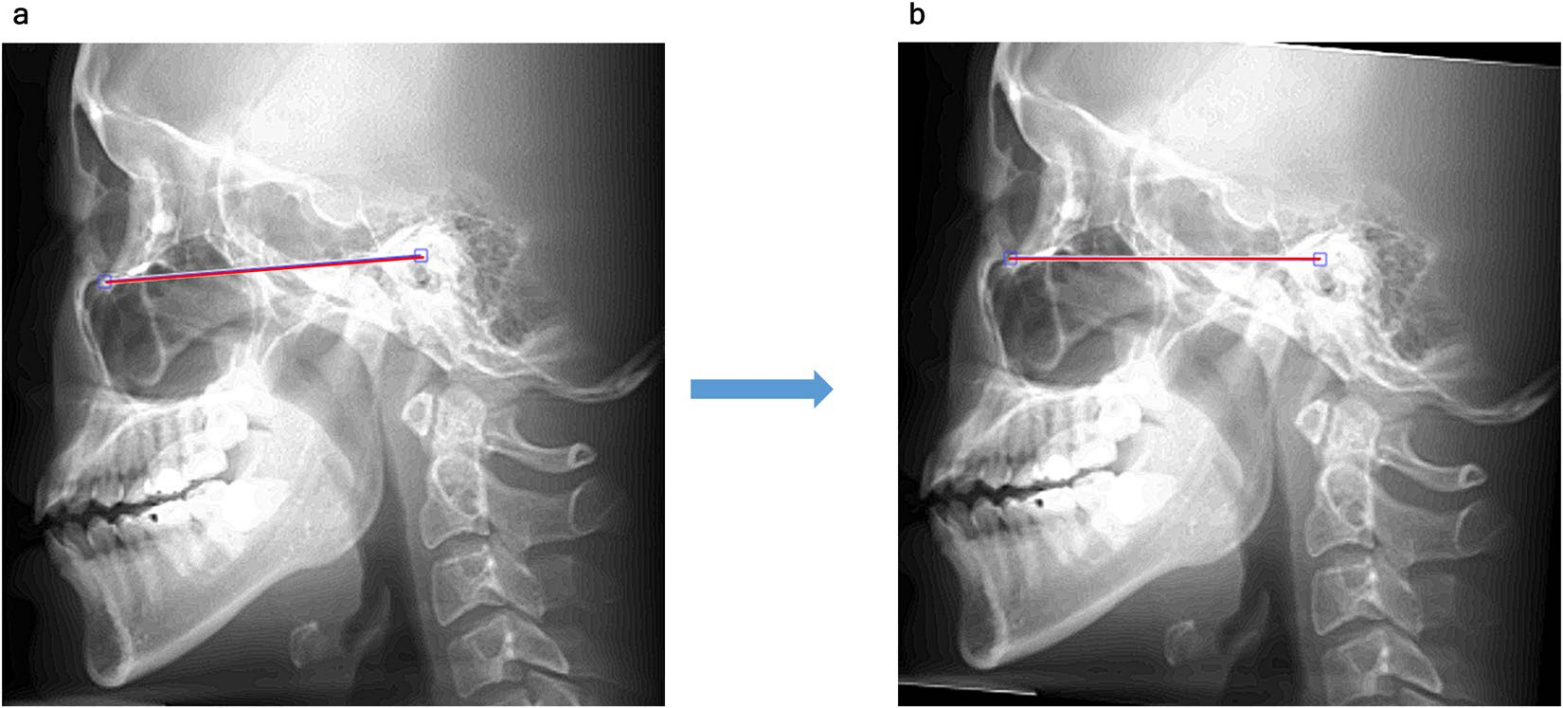
Image reorientation (sagittal). (**a**) Using the average intensity projection image in the sagittal direction, a Frankfort plane (red segment) was drawn passing through the 2 points defined by the inferior margin of the orbit and the upper margin of the external auditory meatus. (**b**) The entire image was rotated around the center of the two points for the Frankfort plane to be parallel with the floor.
- The entire 3D image was rotated around the center of the 2 points for the Frankfort plane to parallel with the floor (Fig. 2b). This procedure aligns the position of the head in the image for panoramic radiography.

1. Image normalization:
  The intensity value of the image was normalized between 0 and 1 by using the Equation (1) below.
  1 $$\mathrm{Normalized}\,\mathrm{image}=\frac{\mathrm{Original}\,\mathrm{image}+m}{M+m}$$
  where *m* indicates minimum pixel intensity and *M* refers to maximum pixel intensity.
2. Reorientation (rotation of the Frankfort plane to be parallel with the floor):

- In the average intensity projection image in the sagittal direction, a Frankfort plane was drawn passing through the inferior margin of the orbit and the upper margin of the external auditory meatus (Fig. 2a).

3) Removal of the facial bone (middle and anterior), cervical vertebrae, and temporal bone superior to the condylar head:

The regions impeding segmentation were removed for the stable segmentation of the mandible.

**Figure 3:**
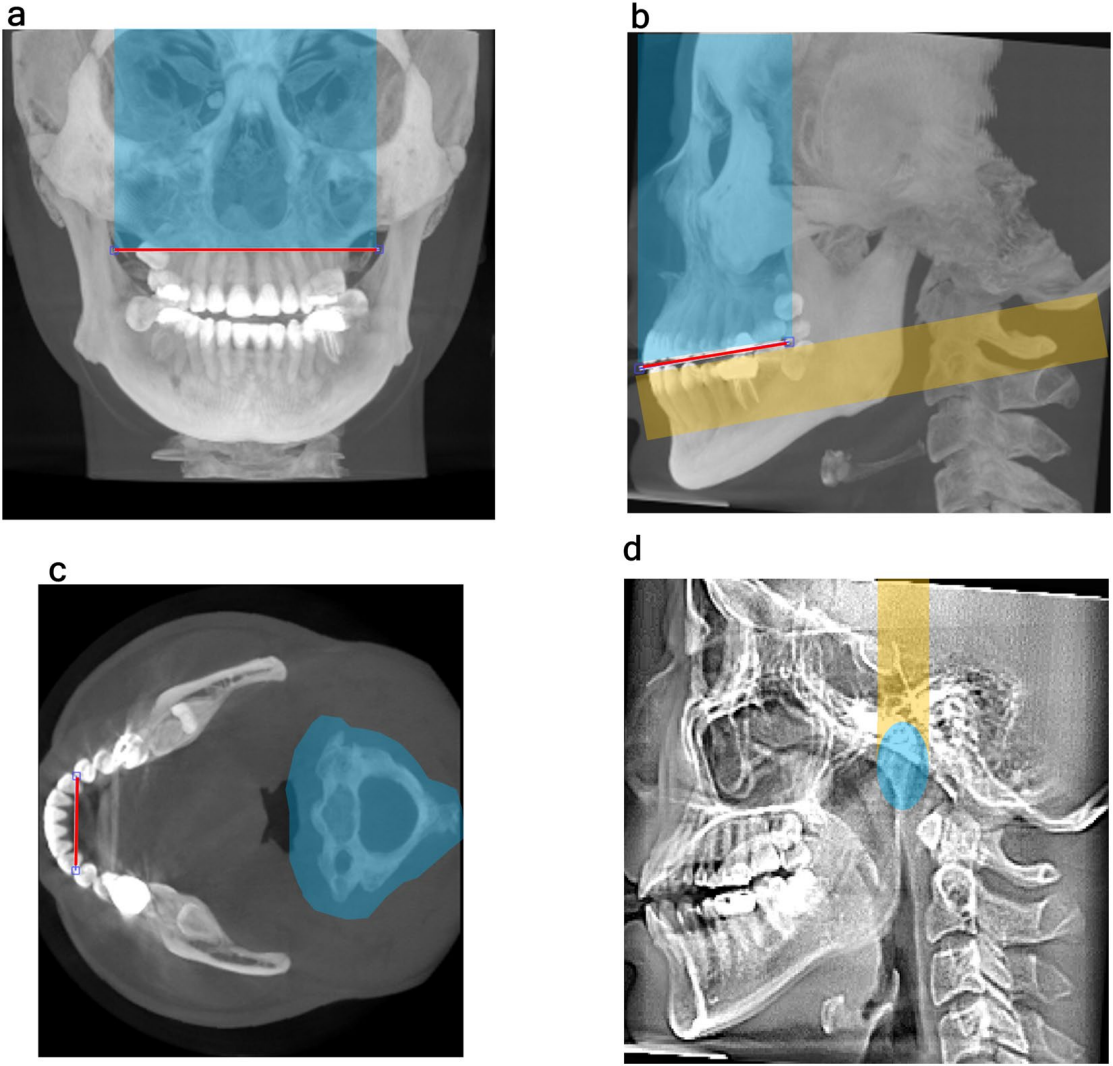
Removal of disturbing regions and location of the distal contact point of the canine. (**a**) In the coronal MIP images, after manually selecting 2 points that met both rami past the root apex of the maxillary central incisor, the blue area superior to the line connecting the 2 points was removed from the entire image. (**b**) In the sagittal MIP image, after manually selecting 2 points that passed through the occlusal plane, the blue area superior to the line connecting the 2 points was removed from the entire image. (**c**) In axial MIP images from the occlusal plane to 2 cm (mean tooth length) below the occlusal plane, the cervical vertebrae posterior to the mandible were removed from the entire image after being magnified to 1.5 times their original size (blue area). The bilateral distal contact points of the canines were manually selected. (**d**) In the sagittal average intensity image, the upper portions of the bilateral condyles were positioned and removed using an eclipse measuring 15 mm × 21 mm. MIP, maximum intensity projection.
- Removal of the anterior facial bone (sagittal direction).

- Removal of the midfacial bone (coronal direction).
  In the coronal maximum intensity projection (MIP) image, after manually selecting 2 points that met both rami passing through the root apex of the maxillary central incisor, the area superior to the line connecting the 2 points was removed from the entire 3D image (Fig. 3a).

In the sagittal MIP image, after manually selecting 2 points passing through the occlusal plane (the anterior point was the edge of the first incisor and the posterior point was the posterior margin of the crown of the second molar), the area superior to the line connecting the 2 points was removed from the entire 3D image (Fig. 3b).

c. Removal of the cervical vertebrae (axial direction).
  In the axial MIP image from the occlusal plane to 20 mm (average tooth length) below the occlusal plane (in Fig. 3b), the cervical vertebrae were segmented automatically using their location relative to the mandible and magnified to 1.5 times their original size (Fig. 3c). This magnified image was removed from the entire 3D image.
d. Removal of the temporal bone superior to the condylar heads.

In the sagittal average intensity projection image, the upper portion of the bilateral condylar heads was positioned and removed using an eclipse measuring 15 mm × 21 mm (Fig. 3d).

4) Location of the distal contact points of the bilateral canines: The bilateral distal contact points of the mandibular canines in the above-described axial MIP image were manually located (Fig. 3c). These contact points were the closest points in the mandible corresponding to the middle of the maxillary canines, which were used as reference points to position the focal trough^19^.

#### Binarization

Otsu’s method was used to obtain the threshold for the automatic segmentation of the CT data^20,21^. This algorithm assumes that the image consists of pixels following a bi-modal histogram (foreground and background pixels), and it then calculates the optimum threshold separating the 2 classes so that their intra-class variance is minimal^22^. We removed the regions impeding segmentation before the binarization process because CBCT images have some inaccurate gray values that disturb the bi-modal distribution of the soft tissue and bone^23^.

**Figure 4:**
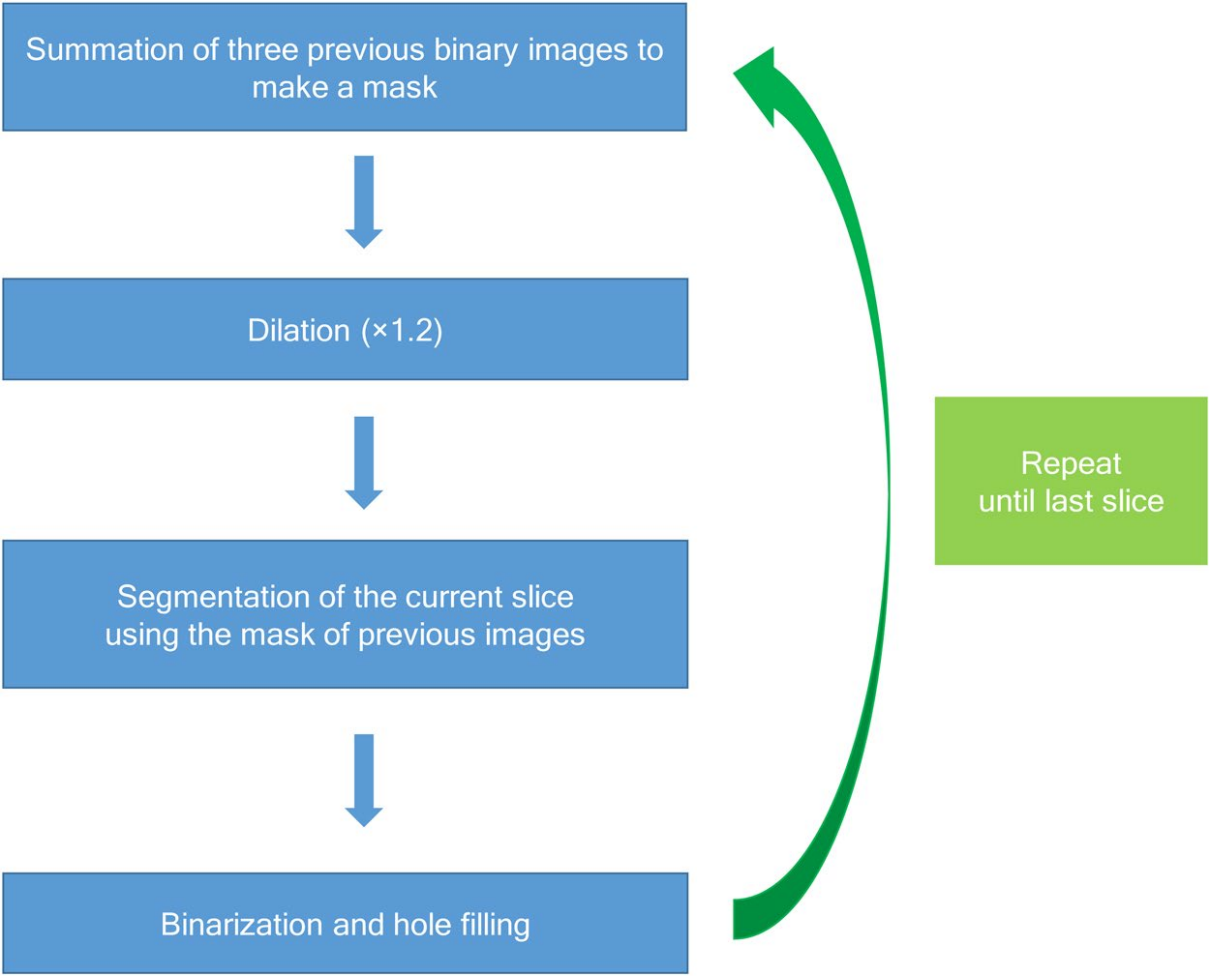
A cumulative binarization algorithm for mandible segmentation. This algorithm summed the 3 previous binarization images to create a mask for the segmentation of the current slice.

We also used a new algorithm for cumulative binarization (Fig. 4), which summed the 3 previous results to optimize the binarization process. This summed image was used as a mask by being magnified to 1.2 times its original size and applied to the current slice, which was followed by the binarization process using Otsu’s method. This led to better stability than can be obtained using direct binarization of each slice because this algorithm refers to previous anatomic structures that continue smoothly into the current slice.

**Figure 5:**
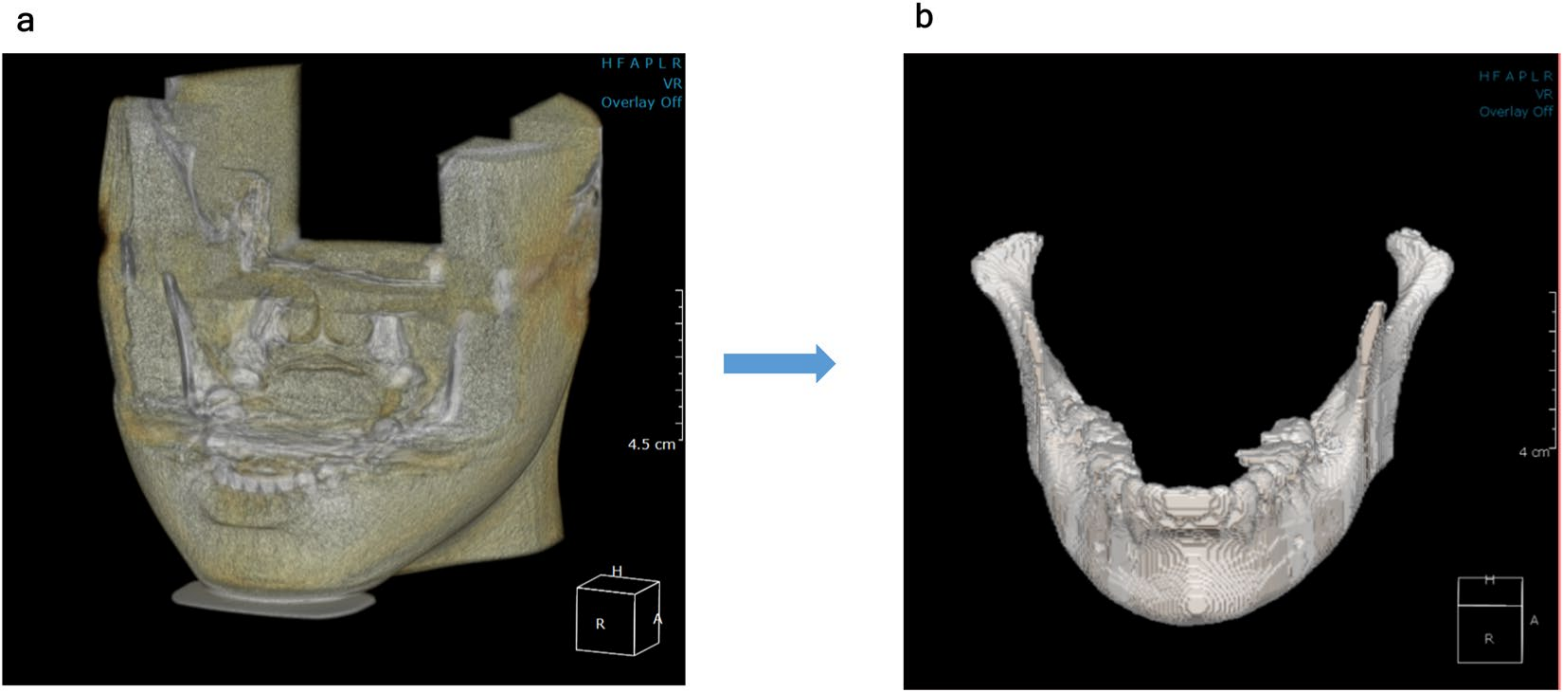
Binarization of the mandible. (**a**) Representation of the processed 3D CBCT image. (**b**) The 3D images were binarized using Otsu’s method and the cumulative binarization algorithm until reaching the upper points of the condylar head obtained in the previous step of removing the upper portion of the bilateral condylar heads (Fig. 3d). 3D, 3-dimensional; CBCT, cone-beam computed tomography.

The 3D images (Fig. 5a) were binarized (Fig. 5b) until reaching the upper points of the condylar head obtained in the previous step of removing the upper portion of the bilateral condylar heads (Fig. 3d).

#### Obtaining the average arch shape by superimposing the segmented images

1. Arch generation in the axial direction:
  Because the focal trough of the available panoramic machines showed a transition between the dental arch and the more laterally positioned ascending ramus^8,9,15,24^, the segmented 3D images (Fig. 6a) were projected in the axial direction using the MIP by selecting the dental arch and the posterior mandible containing the ramus and condyle (Fig. 6b). Only the object with maximum size was left in case the temporal bone was included in the result.

**Figure 6:**
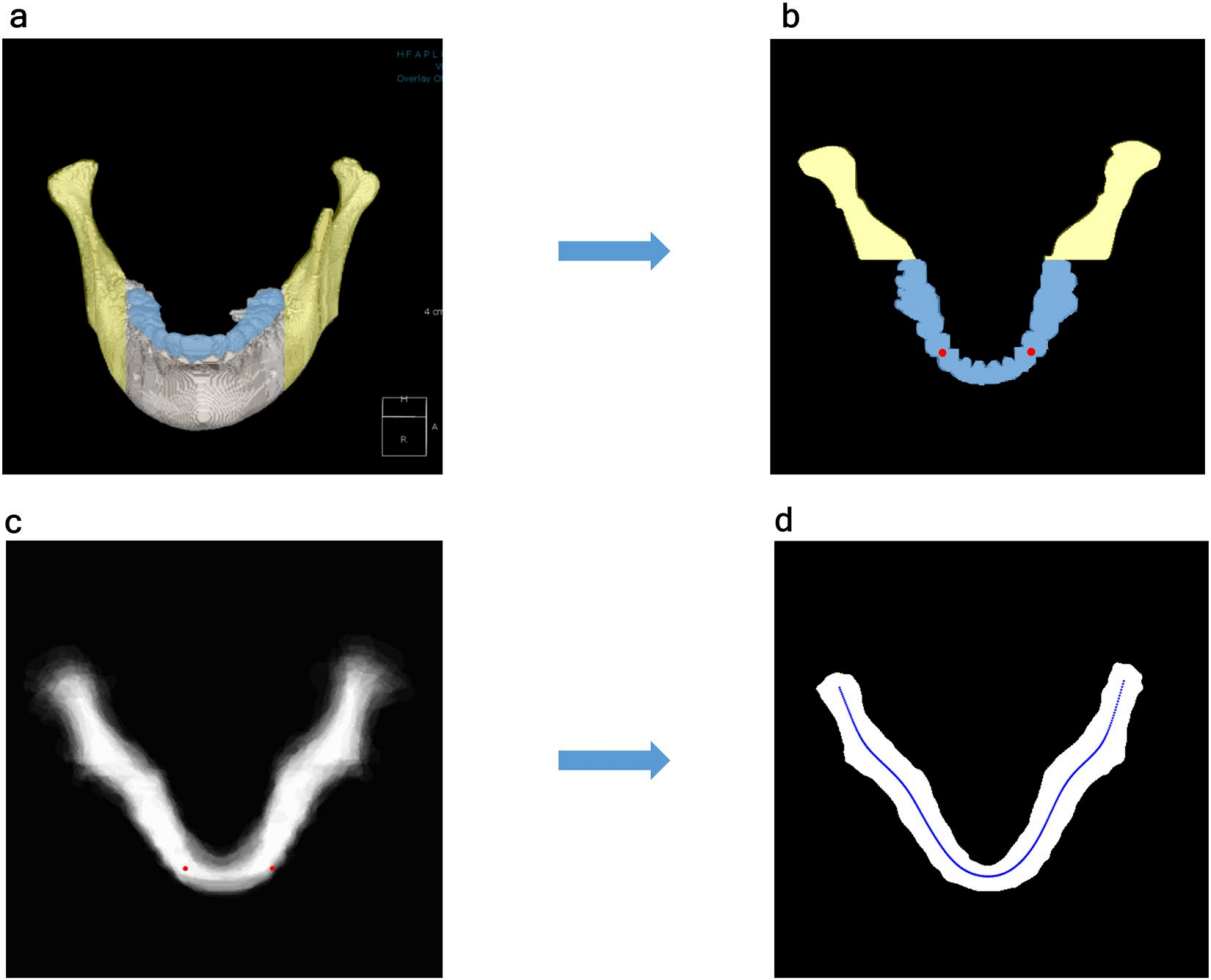
The process of obtaining the average arch model using the segmented 3D image. (**a**) Segmented 3D mandible. (**b**) Axial MIP image created by selecting the dental arch and the posterior mandible containing the ramus and condyle in the segmented 3D image. The yellow area represents the ramus and condyle, whereas the blue area represents the dental arch. (**c**) Thirty MIP images were superimposed on the center of the bilateral distal contact points of the canines (red points). (**d**) The added images were binarized using a threshold value of 0.47 to obtain the average arch shape. A central line represents the center of the average arch shape. 3D, 3-dimensional; MIP, maximum intensity projection.
2. Composite portraiture of 30 patients’ mandibular arches:

Thirty MIP images were superimposed on the center of the bilateral distal contact points of the canines (Fig. 6c).

The added images were binarized using a threshold value of 0.49, which gave the closest result to the average area (2413.30 mm^2^) of 30 arches, to obtain the average arch shape (Fig. 6d). This threshold will become closer to the average threshold value of 0.5 as more data are used.

#### Deriving the center, boundaries, and minimum thickness of the focal trough

The curve fitting procedure was performed automatically by fitting smoothing splines after averaging the right and left side of the curves. This averaging procedure is needed because the panoramic focal trough has a symmetrical shape.

**Figure 7:**
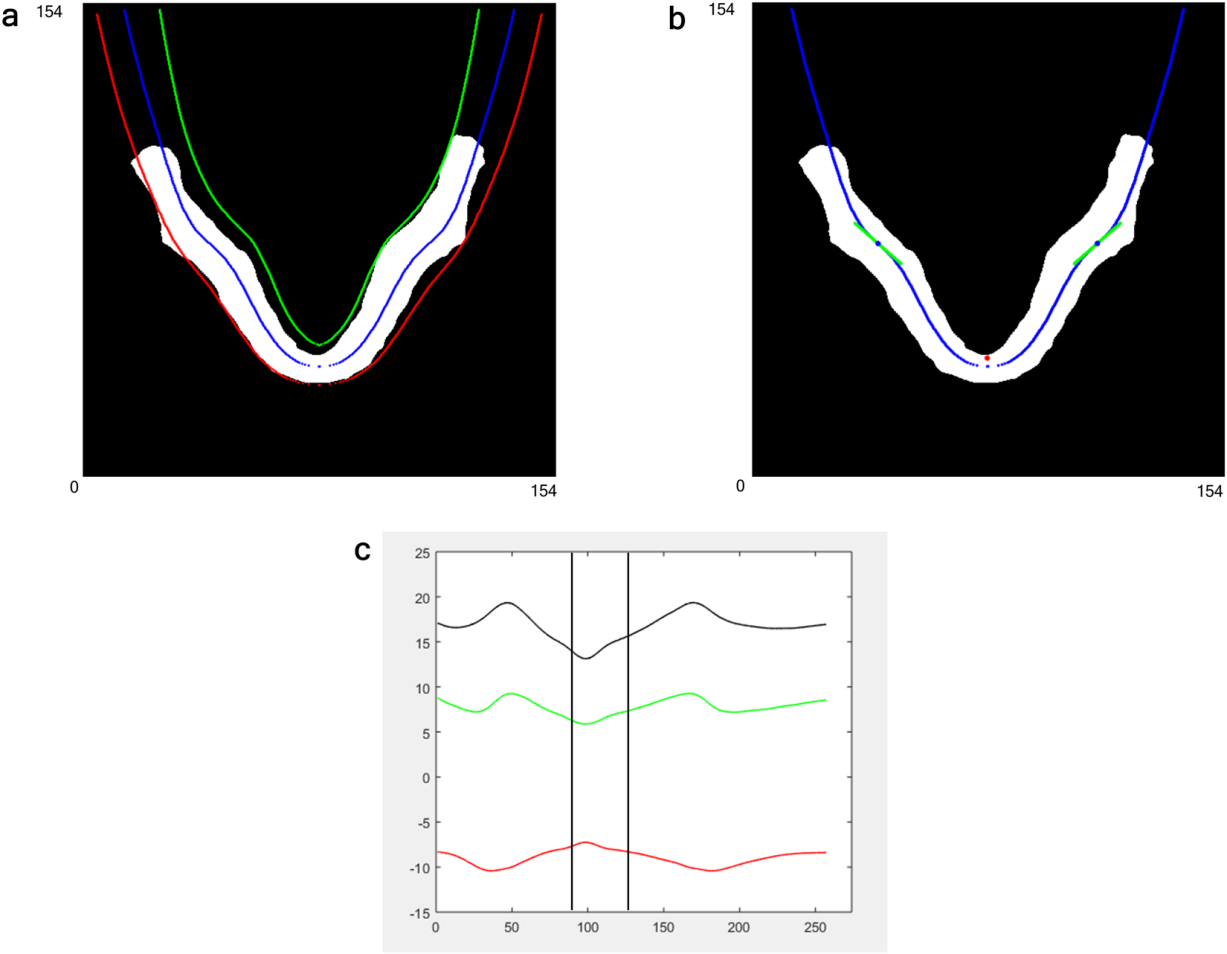
Center, boundaries, and minimum thickness of the panoramic focal trough. (**a**) The fitted center and boundaries of the focal trough using the average arch shape. Each blue, red, and green curve represents the center, buccal, and lingual boundaries of the focal trough, respectively. (**b**) The location (blue points) and slope (green segments) of the transition were obtained using the second and first derivatives of the center of the focal trough, respectively. The red point represents the center of the bilateral contact points of the canines. (**c**) Buccal and lingual boundaries of the focal trough were stretched for the central of the focal trough to become a straight line for obtaining the minimum focal trough thickness. The range of the x-axis is the length of the mandible. The black curve represents the distance (thickness) between the 2 boundaries. Two black vertical lines represent the position of the distal contact points of the bilateral canines. Unit: mm.

1. Center of the focal trough:

The center of the focal trough (solid curve in Fig. 7a) was obtained by fitting a curve to the center (solid line in Fig. 6d) of the average arch shape.

2) Boundaries of the focal trough:
  The boundaries of the focal trough (Fig. 7a) were obtained by fitting curves to each buccal and lingual edges of the average arch shape.
3) Minimum focal trough thickness:

The buccal and lingual boundaries of the focal trough were stretched for the center of the focal trough to become a straight line in order to obtain the focal trough thickness (Fig. 7b). This thickness can be thought of as a minimum, because it contains the border of the average arch shape, and recent panoramic machines have a wider focal trough than the average arch^8,9^.

#### Obtaining the location and gradient of the transition between the dental arch and the ramus

To conceptualize the transition between the dental arch and the ramus, the location and slope of the transition were defined. The location and slope of the transition were obtained using the second and first derivatives of the center of the focal trough, respectively.

### Statistical analysis

To validate the automatic segmentation algorithm, the mandibles were manually segmented and transferred to STL (standard triangulated language) using the OnDemand 3D application (Cybermed, CA, USA) by one radiologist. The STL files were registered to automatic segmentation results using the intensity based ‘imregister’ function of MATLAB (‘multimodal’, ‘InitialRadius = default/3.5, ‘MaximumIterations’ = 300) because the transferred STL files have no dimensional information. Both segmentation results were compared using dice coefficients^25^. The intraclass correlation coefficients (ICCs) of the intra and inter-examiner were used to evaluate the coordinates for removing the facial bone and upper portion of the condylar heads, and to locate the distal contact points of the bilateral canines. Dice coefficients were also calculated to evaluate the similarity of the segmented arches between the repeated experiments.

## Results

The dice coefficients of the segmented arches between the manual and automated method were high (0.91 on average). The inter-examiner (0.75) and intra-examiner (0.78) ICCs of coordinate determination were good and the dice coefficients between observers were also high (0.92 on average, Table 2). The entire list of the coordinates and dice coefficients can be found as Supplementary Table S1-S5. The center of the focal trough showed a transitional arc shape between the dental arch and the ramus (Fig. 7a). The transition was located 37.25 mm laterally and 37.03 mm posterior to the bilateral distal contact points of the canines. The slope of the transition was 41.24° (Fig. 7b). The breaks and fourth-order polynomial coefficients of the smoothing splines can also be found in Supplementary Table S6. The minimum thickness of the focal trough of the average arch shape was 13.09 mm in the anterior mandible (from the incisor to the canine) and 19.25 mm in the posterior mandible (from the first premolar to the condylar head). This thickness tended to increase from anterior to posterior, but did not increase proportionally (Fig. 7c).

**Table 2:** Coordinates and intraclass correlation coefficients of 10 points used in this study.

Points	Coordinates	Mean ( ± SD)	ICC Inter-examiner	ICC Intra-examiner, observer 1	ICC Intra-examiner, observer 2
Anterior point of Fig. 2a	x	177.11 (11.29)	0.32	0.93	0.72
	y	41.30 (10.28)	0.93	0.79	0.93
Posterior point of Fig. 2a	x	50.46 (10.96)	0.97	0.76	0.81
	y	26.40 (10.56)	0.88	0.60	0.93
Right point of Fig. 3a	x	197.90 (3.88)	0.17	0.18	0.62
	y	86.89 (8.41)	0.58	0.19	0.58
Left point of Fig. 3a	x	58.66 (4.26)	0.94	0.41	1.00
	y	86.45 (8.52)	0.92	0.17	0.76
Anterior point of Fig. 3b	x	94.36 (10.86)	0.26	0.79	0.65
	y	115.22 (8.42)	0.75	0.95	0.83
Posterior point of Fig. 3b	x	16.75 (10.60)	0.77	0.93	0.97
	y	131.12 (10.12)	0.82	0.83	0.95
Upper point of Fig. 3c	x	156.28 (3.35)	0.91	0.88	0.43
	y	219.5 (11.04)	0.96	0.98	0.85
Lower point of Fig. 3c	x	105.52 (3.60)	0.44	0.90	0.78
	y	221.9 (10.80)	0.99	0.94	0.96
Right point of Fig. 3d	x	143.38 (10.54)	0.94	0.92	0.99
	y	56.35 (9.69)	0.86	0.95	0.93
Left point of Fig. 3d	x	149.13 (11.18)	0.82	0.69	0.88
	y	56.84 (9.77)	0.76	0.63	1.00

ICC; intraclass correlation coefficient.

## Discussion

Patient positioning is crucial for the standardization of panoramic radiography. It has been reported that distorted and blurred images can occur when the patients’ head moves sagittally about 1 cm antero-posteriorly and 3 cm laterally from the ideal position^26^. As patient positioning is critical for standardizing panoramic images, the standard focal trough designed to fit the average anatomical structure is also important. The panoramic focal trough is designed to accommodate the average arch shape, comprising the tooth-bearing area of the dental arch and the ascending ramus and condyle of the mandibular arch^8,9,24^. If there is a gap between the average arch shapes assumed by various brands of panoramic equipment, geometrical discrepancies between the focal trough and the patient’s true arch may affect the diagnostic accuracy, as the anatomical structures may be distorted or not clearly visible^27–30^.

Despite the diversity of arch shapes and dimensions according to race, sex, and age group^4–7^, current panoramic imaging technology has not reached to point of being able to consider the individual arch shape or to incorporate this diversity into standard focal troughs^8–10^. Therefore, obtaining standard focal troughs using average arch shapes is important for improving the image quality and diagnostic accuracy of panoramic radiography for the following reasons: (1) A standard focal trough that is specific to each race, sex, and age group increases the likelihood of obtaining clearer images in the majority of patients. (2) The standard deviation of each group can be measured only after the average arch shape has been established. Widening the thickness of the focal trough to include these deviations could be a practical way to improve image quality for patients with various arch forms. (3) The use of different focal troughs in different pieces of panoramic radiographic equipment might cause errors in the interpretation of lesion size and characteristics on initial and follow-up panoramic radiography.

Meanwhile, arch studies in the dental field have mainly focused on the dental arch, with little attention paid to the posterior mandible, which includes the ramus and condyle^16,17,31^. A previous study, in which a panoramic focal trough was created for 6-year-old children, used the dental arch only^7^. In a previous study, average mandibular widths were analyzed using submentovertex radiography in the ramus region;^15^ however, separate shapes of the dentition and posterior mandible were obtained instead of an average arch shape. This manual method using 2D images also had limitations in producing an accurate result owing to overlapping structures. In order to obtain an accurate standard focal trough, an average arch shape should be obtained from 3D images.

Methods for obtaining average 3D skull models have been introduced^32–35^. However, those studies used complex statistical methods in which the images were deformed through scaling, rotation, and non-rigid registration without fixed references. This study presents a new image processing method to acquire a standard focal trough directly from 3D CBCT data using the canines as reference points. This study also eliminates manual adjustment of the binarization coefficients by applying Otsu’s method for automatic segmentation. The accuracy of the binarization could be improved by confining the area for the process (removing impeding areas) and referring to continuations of previous anatomic structures (cumulative binarization). The method used in this study is more advantageous for constructing a standard focal trough using big data because it takes less time and effort than previous manual methods of drawing outlines since only 10 points need to be located.

The focal trough could be obtained automatically by fitting smoothing splines to the center and boundaries of the average arch. In addition to the shape and thickness of the focal trough, the mandibular arch shows a transitional arch shape between the dental arch and the ramus, which modern panoramic machines have tried to reproduce^8,9,24^. Because arch shape and dimension differ according to race, sex, and age group^4–7^, the shape of this transition also must be different between each group. If a panoramic machine fails to incorporate this transition in its focal trough, it creates distortions and an unacceptable lack of sharpness on the projected image^8^. In this study, the location and slope of this crucial point was conceptualized and obtained for the first time by selectively assembling the dental arch and the ramus, which can be a useful reference for comparing and classifying various arch shapes.

This retrospective study presented a convenient way of obtaining a standard arch shape from 3D CBCT data using a customized algorithm. The most obvious limitation of this study is its sample size. Only 30 patients’ data were used because this study focused on the introduction of a new automatic method. If this algorithm is applied in a big data study and the established focal trough is integrated in the panoramic machine, it will become possible to select a standard focal trough suitable for a patient’s race, age, and gender, enabling a clearer image with constant distortion of the anatomical structures. Panoramic reconstructions from CBCT or MSCT (multi-slice computed tomography) use the same boundary information as standard panoramic radiography. Since the central plane and boundaries of the arch shape are obtained during this process, the development of an automated algorithm for obtaining panoramic reconstruction images from CBCT or MSCT data will also be possible.

## Electronic supplementary material

Supplementary table S1

Supplementary table S2

Supplementary table S3

Supplementary table S4

Supplementary table S5

Supplementary table S6

## Data Availability

All data generated or analyzed during this study are included in this published article (and its Supplementary Information files).
